# Modifiable Factors Underlying Caregivers’ Psychological Support Needs in Pediatric Disability: Through the Lens of Psycho-Behavioral and Social–Environmental Interactions

**DOI:** 10.3390/healthcare13060625

**Published:** 2025-03-13

**Authors:** Hongying Zheng, Mei Sun, Anni Wang, Qi Tang, Yaping Zhang, Jun Lu, Gang Chen

**Affiliations:** 1 School of Public Health, Fudan University, Shanghai 200032, China; 20111020062@fudan.edu.cn (H.Z.); sunmei@fudan.edu.cn (M.S.); tangqi@fudan.edu.cn (Q.T.); 2 China Research Center on Disability, Fudan University, Shanghai 200032, China; 3 School of Nursing, Fudan University, Shanghai 200032, China; wanganni@fudan.edu.cn; 4 Occupational Medicine Center, Shanghai Institute of Occupational Disease for Chemical Industry, Shanghai 200041, China; zhyp0827@163.com

**Keywords:** children with disabilities, caregivers, psychological support needs, need satisfaction, individual–social interaction

## Abstract

**Background/Objectives**: Childhood disability is a major stressor for caregivers. There are many problems and challenges in relation to satisfying the psychological support needs of caregivers. This study aims to explore the modifiable psycho-behavioral and social–environmental factors of psychological support needs and need satisfaction, their interaction effects, and their configuration paths. **Methods**: This was a cross-sectional survey of 363 caregivers using instruments such as the psychological support needs subscale of the Care Needs Assessment Tool for Children with Disabilities. Multivariable logistic regression with interaction terms and crisp-set qualitative comparative analysis were performed. **Results**: The overall rate of psychological support needs was 78.29%, and the overall need satisfaction was 49.94%. Multivariable logistic regression analysis showed that the caregiver’s need was mainly influenced by anxiety, while need satisfaction was primarily predicted by public policy support and social attitudes. There was an interaction effect between caregiver anxiety and social attitude on need satisfaction. Qualitative comparative analysis indicated that six paths were identified as potentially leading to high levels of psychological support need, while five paths were associated with low levels of need satisfaction. **Conclusions**: The psychological support need rate among caregivers was high, and the level of need satisfaction was low. There were significant differences in psychological support needs and need satisfaction among different psycho-behavioral and social–environmental characteristics. It is recommended that caregivers with negative emotions be given more attention. The enhancement of social attitudes and the adoption of more supportive policies will contribute to the improvement of need satisfaction.

## 1. Introduction

The World Report on Disability [1] estimated that there were globally about 93 million children with disabilities (CWDs) aged 0–14 years, accounting for about 5.1%. The Second China National Sample Survey on Disability [2] showed that there were already 5.04 million CWDs nationwide in 2006, accounting for 1.6% of the total number of children and 6.1% of the total number of disabled individuals. Among these, children with intellectual disabilities accounted for a relatively substantial proportion, reaching 1.749 million. The second-largest group comprised children with multiple disabilities, with a count of 1.435 million, followed by children with physical disabilities (0.899 million). Conversely, the number of children with mental disabilities was the lowest, approximating 0.155 million. In sequence, the groups with relatively fewer numbers were children with hearing disabilities (0.205 million), children with visual disabilities (0.241 million), and children with speech disabilities (0.369 million). The risk of congenital and acquired disabilities continues to increase, and the number of CWDs in China will continue to climb due to congenital defects, diseases, and accidents. Childhood disability is a major stressor for caregivers. They suffer from significant psychological trauma and experience anxiety [3], depression [4], helplessness, post-traumatic stress disorder [5], and many other negative psychological aspects [6,7,8], which may persist even years after the child’s diagnosis.

Considering the many psychological problems currently affecting caregivers, focusing on psychological support needs can help caregivers and their families better fulfill their caregiving tasks [9] and better achieve the full rehabilitation of CWDs [7,8]. Moving beyond the traditional terminology associated with religious or pastoral care, psychological support has been described as “the provision of a ministry of presence and emotional support to individuals or groups”. Thus, in this study, we use the term “psychological support” as an integral component entailing a psychological presence and comfort, establishing a psychologically nurturing environment, and other actions. This conceptualization of psychological support can be applied to all healthcare providers, from medical clinicians to psychological counselors, as well as their family, relatives, friends, and peer support groups and the public.

However, there are many problems with the psychological support needs and need satisfaction of caregivers [10,11]. A previous study found a high rate of psychological support needs among caregivers of CWDs (75%) [8], especially for professional psychological support programs. In addition, the impact of time constraints; financial constraints; a lack of professional healthcare providers to provide support; difficulty in making appointments for psychological health services; and misperceptions of caregivers, such as not perceiving psychological problems as serious enough to require help [8] results in a low level of satisfaction for psychological support needs. Prejudice, discrimination, and rejection can be shown by the public toward CWDs, and caregivers of CWDs are often reluctant to seek psychological support outside of the family due to stigma and illness [12]. As we all know, the need for psychological support remaining unmet is harmful to both children and their caregivers. It not only seriously affects the physical and mental health of caregivers but is also detrimental to service utilization, healthy development, and social participation among CWDs and can lead to the deterioration of family functioning and increase the burden on families [8,11].

**Figure 1:**
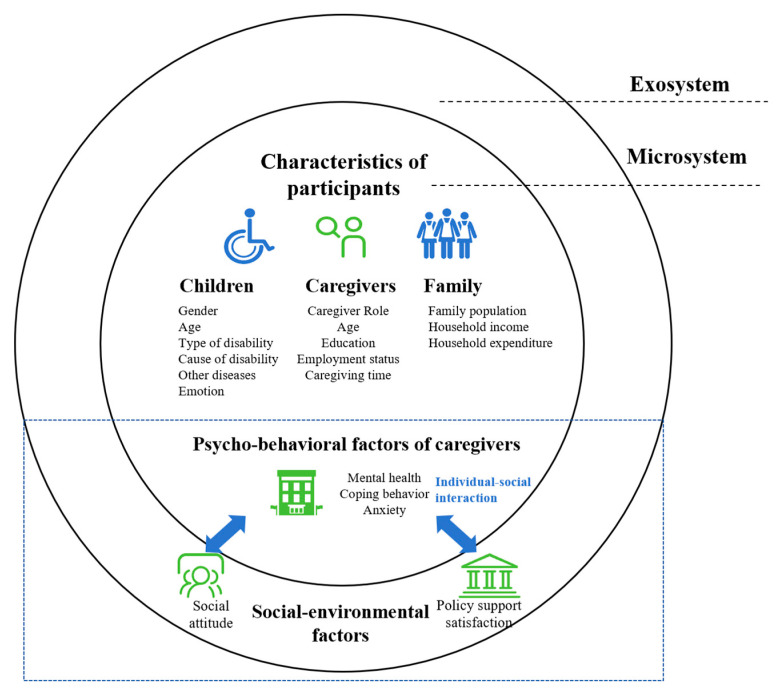
Schematic of modifiable psycho-behavioral and social–environmental factors.

According to the International Classification of Functioning, Disability, and Health (ICF) [13] and Ecological Systems Theory (EST) [14], psychological support needs and need satisfaction can be influenced by both individual (microsystem) and environmental (exosystem) factors. Furthermore, the social determinants of health (SDH) model [15] indicates that a multitude of factors influence an individual’s health. From the inside out, the first level comprises individual characteristics such as age and gender. The second level comprises individual behavioral and lifestyle characteristics. Other outer layers include community factors, social structural factors, and macro-environmental factors. Previous studies [9,12,16,17] have identified individual factors such as disability type, caregiver role, and modifiable psycho-behavioral and social–environmental factors (Figure 1). The psycho-behavioral factor is a broad concept that refers to the caregiver’s psychological state, coping behaviors, etc. Social–environmental factors are numerous and complex. Social–environmental factors [1,13] are the main cause of caregivers having unequal opportunities in society, including social attitudes such as social friendliness or social exclusion, public policy support, etc. In contrast to demographic characteristics, modifiable factors possess both alterable and intervenable properties that warrant particular attention. From an individual–social interaction perspective, individuals exist within specific environmental systems that interact with their psycho-behavioral states, thereby influencing personal development. However, limited studies have focused on the valuable interaction between individual psycho-behavioral factors and social–environmental factors. This study aims to address the following research questions: (i) Which modifiable psycho-behavioral and social–environmental factors are the dominant ones? Are there interaction effects between these factors, and (ii) what factor configurations can lead to high levels of needs and low levels of need satisfaction? What are the configuration pathways? The aim of this study was to examine the modifiable psycho-behavioral and social–environmental factors related to psychological support needs and need satisfaction, as well as their interaction effects and configuration paths.

## 2. Materials and Methods

### 2.1. Study Design

This study was a cross-sectional survey reported strictly in accordance with the Strengthening the Reporting of Observational Studies in Epidemiology checklist for cross-sectional studies [18].

### 2.2. Participants

In this study, caregivers were recruited using the convenience sampling method from December 2019 to January 2020 and from August 2020 to September 2020 in 12 rehabilitation institutions and eight communities in eight districts of Shanghai, China.

The inclusion criteria for caregivers of CWDs are as follows: (a) child’s father or mother; (b) assuming primary care responsibilities for the child; (c) being familiar with the child’s health status; (d) caring for a child with disabilities or developmental delays; (e) caring for a child aged 0–18 years old; (f) providing informed consent. The exclusion criteria for caregivers of CWDs are as follows: (a) refusal to cooperate due to privacy concerns, time constraints, lack of understanding, etc.; (b) inability to complete the questionnaire either through written or one-on-one inquiries.

The sample size was calculated separately from the perspectives of psychological support need and need satisfaction using the following formula.

(1) $$N=\frac{{Z^{2}}_{\alpha /2}\pi (1-\pi )}{\delta^{2}}$$

The allowable error was set to no more than 0.05, and α was taken as 0.05. Considering the questionnaire return rate and validity, the sample size was increased by 20%. Based on the previous literature [8,16], the calculated sample size of caregivers from the perspectives of psychological support need and need satisfaction was 346 and 192, respectively. Therefore, at least 346 caregivers had to be included in this study for analysis. The final sample comprised 363 caregivers of CWDs.

### 2.3. Measures

#### 2.3.1. Independent Variables

##### Individual and Family Characteristics

The general information questionnaire encompassed both individual and family characteristics. Children’s demographic characteristics included age, gender, disability type, and cause of disability. The characteristics of caregivers consisted of their role, age, employment status, and years of caregiving. Family characteristics covered family size, household expenditure, and household income.

##### Modifiable Psycho-Behavioral Factors of Caregivers

The modifiable factors were ascertained through a rigorous analysis of the literature. Frequently mentioned modifiable psychological factors included caregiver anxiety, mental health status, and coping behaviors.

- The Generalized Anxiety Disorder 7-item Scale

The Generalized Anxiety Disorder 7-item Scale (GAD-7) [19], developed by Spitzer in 2006, was used to assess individuals’ anxiety symptoms. The GAD-7 consists of seven items and adopts a Likert 0–3 four-point scale. Total scores range from 0 to 21, with higher scores reflecting greater anxiety severity. A score ranging from 0 to 4 indicates the absence of anxiety symptoms, while a score of above 5 indicates the presence of such symptoms. The Chinese version of the GAD-7, translated by Li [20] in 2010, was demonstrated to have good reliability and validity among general hospital outpatients.

- The 12-item Short-Form Health Survey–Mental Component Summary

The 12-item Short-Form Health Survey (SF-12) is widely used to measure health-related quality of life (HRQoL). The SF-12 [21] comprises 12 items and two types of composite indicators: the Physical Component Summary (PCS) score and the Mental Component Summary (MCS) score. Lam [22] translated the scale and validated the equivalence of the SF-12 in the Chinese population, which has good reliability and validity. The previous study [23] found that the MCS norm of Chinese residents was 49.9. A higher MCS score indicates better mental health, while a score below the MCS norm indicates poorer mental health.

- The Coping Health Inventory for Parents

The Coping Health Inventory for Parents (CHIP), developed by McCubbin [24], is commonly used to investigate the coping behaviors of caregivers of CWDs. The Chinese version of the CHIP instrument, translated by Li [25], comprises 45 self-report items and is divided into three subscales. The content validity index (CVI) was 0.82, and the Cronbach’s α coefficient of the three subscales was 0.92, 0.80, and 0.76. A previous study [26] revised this instrument, and it is widely used in evaluating the coping behaviors of caregivers of CWDs. The revised version of the CHIP instrument measures the frequency and usefulness of different coping behaviors. In this study, parents were asked whether they used these coping behaviors or not. Owing to the lack of a norm, coping behavior scores above the mean indicate the use of positive behaviors.

##### Modifiable Social–Environmental Factors

According to the ICF [13,27], social–environmental factors (excluding natural environmental factors) that might influence the participation of CWDs are classified into the following categories: (1) attitudes and (2) services, systems, and policies. To enhance the relevance of social–environmental factors, this study further summarized the existing literature and categorized social–environmental factors into two main aspects: (1) social attitude and (2) public policy support. Given the absence of mature scales and assessment tools for measuring the social environment, self-designed single-item questionnaire entries were developed to measure these social–environmental factors, taking into account the real context in China. Social attitude includes social friendliness and social exclusion. CWDs and their caregivers are in an unequal position in society because of certain barriers, and unfriendly social attitudes may further exacerbate their unequal opportunities. Therefore, this study designed a measurement item for social attitude friendliness, which was evaluated using a three-level Likert method. Public policy support encompasses the support policies for CWDs and caregivers and reflects caregivers’ satisfaction with the allocation of policy support resources. Accordingly, after presenting the existing support policies, a three-level Likert scale was used to measure caregivers’ satisfaction with these support policies.

#### 2.3.2. Dependent Variables

A previous study [28] developed the Care Needs Assessment Tool for Children with Disabilities through a comprehensive literature review, stakeholder interviews, and expert validation. This tool comprises 27 items and five subscales: subscale I—alternative service support needs (4 items); subscale II—professional support needs (7 items); subscale III—financial support needs (5 items); subscale IV—psychological support needs (5 items); and subscale V—information support needs (6 items). In this study, the psychological support needs subscale was used. Each item offers the options “need exists”, “need does not exist”, and “not sure”, which are used to calculate the need rate. Moreover, need satisfaction is assessed using a 0–4 five-point Likert scale, where 0 indicates “not met” and 4 represents ”extremely met”. The need satisfaction is calculated as the ratio of the sum of satisfaction scores to the total score of all existing need items. Previous studies [9,28] showed that the psychological support needs subscale had good reliability. Specifically, the KMO value was 0.790, and the *p*-value of Bartlett’s sphericity test was less than 0.001. Factor analysis revealed one common factor, accounting for 71.02% of the variance, indicating good structural validity.

### 2.4. Procedures

First, based on the degree of cooperation, 12 rehabilitation institutions and eight communities were selected for recruiting caregivers in this study. Subsequently, with the assistance of staff from each rehabilitation institution and community, we obtained preliminary information about the children in the area. For the rehabilitation institutions, we arranged for uniformly trained enumerators to visit the institutions and conduct questionnaire surveys in accordance with the children’s rehabilitation schedules. For the communities, the community staff contacted and assisted the researcher to centralize them relative to a specific survey point where the researcher conducted the questionnaire. Before the investigation, the researcher explained the purpose to the participants using standardized instructions. After the participants signed the informed consent form, participants completed questionnaires either independently or through researcher-assisted interviews. If the participants had any doubts, the researcher responded promptly. After a participant completed the questionnaire, the researcher immediately checked it for completeness. If there were any missing items, the researcher asked the participants to complete them promptly.

### 2.5. Statistical Analysis

#### 2.5.1. Multivariable Logistic Regression Analysis

This study used SPSS 22.0 software and Stata 17 MP software for data analysis. Measurement data were presented as (x ± s), and count data were presented as [*n* (%)]. The two dependent variables of interest in this study were (1) psychological support needs and (2) psychological support need satisfaction. A 60% cutoff value was used to form a dichotomous variable. Univariate analysis was performed using the Chi-square test and nonparametric tests. This study utilized a dichotomous logistic regression model to analyze the influencing factors and examine potential interaction effects.

Individual layer model (model 1): *y = β*_0_
*+ β*_1_*C + β*_2_
*PB + μ* (2)

Social layer model (model 2): *y = β*_0_ + *β*_1_*C* + *β*_2_ *PB* + *β*_3_*SE* + *μ* (3)

Individual–social interaction model (model 3): *y = β*_0_
*+ β*_1_*C* + *β*_2_
*PB + β*_3_*SE + β*_4_*PB × SE + μ* (4)

In these formulas, *y* represented the dependent variable (psychological support need and need satisfaction); *C* denoted a set of control variables; *PB* indicated the psychological-behavioral factors; *SE* represented the social–environmental factors; *PB* × *SE* represented the second-order interaction term formed by psycho-behavioral factors and social–environmental factors.

The study adopted a layer-by-layer approach for analysis. Model 1 included control variables and psychological-behavioral factors. Model 2 further incorporated social–environmental factors based on Model 1. Subsequently, Model 3 further incorporated second-order interaction terms of the modifiable psycho-behavioral and social–environmental factors that were significant in the univariate analysis. The interaction terms were centered to avoid multicollinearity. All variables were tested for multicollinearity, and the variance inflation factors (VIFs) ranged from 1.053 to 1.687, indicating no multicollinearity. A two-tailed *p*-value < 0.05 was considered statistically significant.

#### 2.5.2. Crisp-Set Qualitative Comparative Analysis (csQCA)

csQCA is a method for examining complex causal relationships among cases. It is suitable for handling multi-factor, multi-conditional causalities and can uncover which combinations of conditions result in a particular outcome. While traditionally used for case studies or interview data, csQCA is increasingly applied to questionnaire data analysis. We employed fsQCA 4.1 software for configuration path analyses. Five antecedent variables were examined: mental health (MH), anxiety symptoms (AS), coping behavior (CB), policy support (PS), and social attitudes (SA). Psychological support needs and need satisfaction were considered as the outcome variable. Continuous variables were transformed into binary variables. Then, a necessary condition analysis was performed. Consistency and coverage were calculated to determine whether there was a sufficient or necessary conditional relationship between the antecedent and outcome variables. In constructing the truth table, this study set the consistency threshold at 0.8, the primitive consistency index (PRI) threshold at 0.7, and the case-number threshold at 3. Subsequently, a configuration analysis was performed to ascertain the influence of disparate combinations of antecedent conditions on the outcomes. Based on the parsimonious and intermediate solutions, the core and peripheral conditions were identified, and the final configuration paths were derived.

### 2.6. Ethical Consideration

This study was approved by the Medical Research Ethics Committee of the School of Public Health, Fudan University (Grant No. IRB#2019-10-0782). All participants signed informed consent forms, and the survey was anonymous.

## 3. Results

### 3.1. Characteristics of Participants

#### 3.1.1. Individual and Family Characteristics of Participants

A total of 363 caregivers of CWDs were included in this study. Among the 363 children, 63.36% were male and 36.64% were female. Disability types included developmental delay (10.74%), visual disability (6.61%), hearing disability (15.70%), speech disability (6.89%), physical disability (22.87%), intellectual disability (9.92%), mental disability (14.60%), and multiple disabilities (12.67%). Children in the age group of 0–6 years were predominant, accounting for 55.65%. Among caregivers, fathers accounted for 27.00% and mothers accounted for 73.00%. A total of 61.22% of the caregivers were (30, 40] years old, and most of the caregivers were currently in full-time employment (55.65%). Additionally, 59.94% of the caregivers had caregiving experience of (0, 5] years. Among the family characteristics, 49.59% of the family had 4–5 permanent household members, and 37.78% of the caregivers’ monthly household income was in the range of CNY (0, 5000) (Table 1).

#### 3.1.2. Psycho-Behavioral and Social–Environmental Characteristics

The mean anxiety score of caregivers was 3.88 (SD = 4.43). The mean mental HRQoL score of caregivers was 41.78 (SD = 12.40), which was lower than the mental HRQoL norm of Chinese residents, indicating poorer mental health among caregivers. The mean frequency of caregivers using coping behaviors was 93.15 (SD = 10.38). As for the social–environmental characteristics, only 23.82% of caregivers expressed satisfaction with existing policy support. Additionally, 34.35% of the caregivers perceived the social attitude as unfriendly (Table 1).

### 3.2. Psychological Support Needs Rate and Need Satisfaction

**Figure 2:**
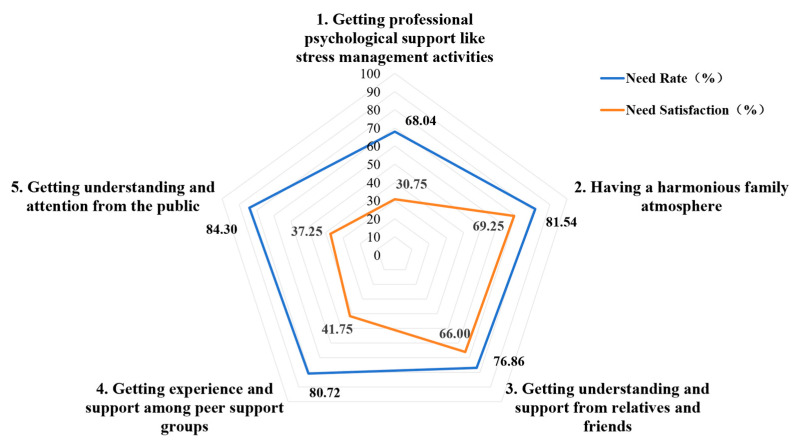
Psychological support need rate and need satisfaction.

The overall rate of psychological support needs was 78.29%, and the overall need satisfaction was 49.94%. The most frequently reported need was “getting understanding and attention from the public” (84.30%), while the least satisfied need was “getting professional psychological support like stress management activities” (30.75%) (Figure 2).

### 3.3. Results of Multivariable Logistic Regression Analysis

#### 3.3.1. Univariate Analysis

Among the 19 factors, statistically significant differences in the psychological support needs were found in the emotional stability of children, caregiver anxiety, caregiver mental health, and policy support (*p* < 0.05). Disability type, cause of disability, caregiver role, anxiety, mental health, coping behavior, policy support, and social attitude significantly influenced need satisfaction (*p* < 0.05) (Table 2).

#### 3.3.2. Multivariable Logistic Regression Analysis for Psychological Support Needs

Model 1 demonstrated the significant direct effects of psycho-behavioral factors, with children’s emotional stability and caregiver anxiety emerging as the key predictors of psychological support needs. Social–environmental factors were incorporated in Model 2. The result demonstrated that the impact of anxiety on psychological support needs remained strong after the inclusion of the variable policy support, but the effect of policy support was not significant. Model 3 represented the full model, including interaction terms. The results of Model 3 indicated that there was no interaction effect, and anxiety remained the main influencing factor. Specifically, a higher level of caregiver anxiety (OR = 1.194, 95%CI 1.065~1.340, *p* = 0.002) was associated with a higher level of psychological support needs among caregivers (Table 3).

#### 3.3.3. Multivariable Logistic Regression Analysis for Psychological Support Need Satisfaction

Model 1 showed that the disability type of children and caregiver anxiety were significant influencing factors. Specifically, a higher level of anxiety (OR = 0.931, 95%CI 0.869~0.997, *p* = 0.041) was associated with lower satisfaction. Model 2 further incorporated social–environment variables, and the results demonstrated that policy support and social attitude were the main influencing factors. Higher public policy support (OR = 1.764, 95%CI 1.148~2.711, *p* = 0.010) and a more friendly social attitude (OR = 1.982, 95%CI 1.362~2.886, *p* < 0.001) were associated with a higher satisfaction of psychological support needs. The interaction terms of psycho-behavioral and social–environmental factors were further added to Model 3. The interaction term of anxiety and social attitude was found to be significant (OR = 0.874, 95%CI 0.783~0.977, *p* = 0.018). This suggests that the impact of caregiver anxiety on the level of need satisfaction was moderated by social attitude, and there was heterogeneity in the effect of anxiety on individual need satisfaction at different levels of social attitude. With continuous improvements in social friendliness, the individual emotional status had an increasingly strong impact on the satisfaction of psychological support needs (Table 4).

### 3.4. Results of csQCA

#### 3.4.1. Necessary Condition Analysis

The consistency values of the necessity analysis for a single condition were all lower than 0.9 (Table 5). Therefore, the six antecedent conditions resulted from multiple factors rather than any single necessary condition.

#### 3.4.2. Configuration Analysis

Five conditional configurations generated six configuration paths for the psychological support needs. The configuration paths were as follows: Path 1 MH*~CB*~SA; Path 2 AS*~PS*~SA; Path 3 MH*AS*CB; Path 4 MH*AS*PS; Path 5 MH*CB*PS; Path 6 ~MH*~AS*PS*SA. Taking Path 1 MH*~CB*~SA as an example, it indicated that individuals with poor mental health, positive coping behaviors, and friendly social attitudes collectively exhibited an elevated need for psychological support. Among these, Path 1, Path 2, and Path 3 were identified as belonging to the group dominated by caregivers’ psycho-behavioral factors. Paths 4 and Path 5 were classified as belonging to the coinfluence-oriented group, while Path 6 was classified as belonging to the group dominated by social–environmental factors.

Concurrently, five conditional configurations generated five configuration paths related to need satisfaction. The following five conditional configuration paths were identified: Path 1 AS*CB*~PS*~SA; Path 2 MH*AS*CB*~PS; Path 3 MH*AS*~CB*PS; Path 4 ~AS*CB*PS*SA; Path 5 ~MH*~AS*~CB*PS*~SA. Among these, Paths 3 and Path 4 were classified as belonging to the coinfluence-oriented group.

In this study, the overall consistency values reached 0.901 and 0.938 (>0.75), respectively. The overall coverage values were 0.645 and 0.284 (>0.25). In terms of case coverage, the psycho-behavioral-factor-oriented group was the highest, followed by the coinfluence-oriented group (Table 6).

## 4. Discussion

Dramatic changes have occurred in China with the implementation of public policies, and the status of psychological support needs and need satisfaction among caregivers of CWDs in these new contexts has also changed. Our main findings can be helpful in discovering and overcoming the risk factors associated with poor psychological support needs, integrating evidence and public policies to enhance the psychological support model, and improving the quality of psychological support.

### 4.1. Current Status of the Psychological Support Need Rate and Need Satisfaction

Existing studies [8,12,16,29] employ diverse needs assessment tools, primarily utilizing total scale scores rather than demand rates and satisfaction levels. Previous research [30] revealed the limited availability of psychological support services for caregivers despite high demands, highlighting an urgent need for intervention. Gilson’s study [8] indicated a 75% psychological support need rate and 58% satisfaction level among mothers of CWDs, consistent with our findings. This study revealed higher psychological support need rates for the public, family, and peer support groups (>80%), while satisfaction levels for professional psychological support and the public remained lower (<40%). Therefore, it is necessary to further ameliorate the segregated, isolated, and alienated environment faced by the caregivers of CWDs; address social discrimination; and foster an equal and friendly social atmosphere. At the same time, it is particularly crucial to expand the channels for psychological consultation and counseling for caregivers and assist them in establishing a robust peer support system.

### 4.2. Influencing Factors of the Psychological Support Need Rate

This study found that among the individual factors, caregivers’ mental HRQoL, caregiver anxiety, and children’s emotional stability were the dominant factors. Caregivers’ mental HRQoL was lower than that of the general population [23], indicating the urgent need for immediate attention and the development of effective interventions for this group. Gloria’s survey [17] of caregivers of individuals with autism spectrum disorders also indicated that caregivers’ mental health has a negative impact on the entire family’s needs, with findings similar to those of this study. Furthermore, caregiver anxiety was a major predictor of psychological support needs. A previous study among caregivers of cancer patients and cancer survivors [31] also indicated that greater anxiety and higher levels of emotional problems were positive predictors of psychological support needs, which in turn could lead to an unfavorable prognosis for family function. Previous research [3] integrated the anxiety situations of caregivers of CWDs and found that the incidence of anxiety symptoms ranged from 21% to 62%, suggesting that attention should be paid to the negative emotions of this group. Additionally, CWDs and caregivers live together in a family environment, and there is an actor–partner interdependence [32] in the emotional expressions of both parties. Therefore, there is an urgent need to enhance caregivers’ awareness of their family’s emotional state and promote the adoption of a family-centered psychological support model [16,33]. Another finding was that addressing public policy support for caregivers was crucial as it was a significant predictor of psychological support needs in univariate analysis. Therefore, there is a clear need to develop public policies for families of CWDs by integrating existing psychological support services, which will ultimately improve outcomes for the entire family.

### 4.3. Influencing Factors of the Satisfaction of Psychological Support Needs

Need satisfaction depends on the supply and utilization of services, and an ideal match between supply and demand can better enhance the level of need satisfaction. Regarding the caregiver role, mothers of CWDs had lower levels of need satisfaction than fathers in this study. Mothers of CWDs play a more important role in child development [4] and have higher expectations for their children than fathers in Chinese families, which is consistent with cultural norms. Hence, it is suggested that we further strengthen psychological support services in the future while focusing on the mental health of mothers of CWDs. Among psycho-behavioral factors, caregivers’ mental HRQoL, anxiety, and coping behaviors were major factors influencing caregivers’ need satisfaction. As we all know, the impact of caregivers’ psychological factors [34] on the need satisfaction cannot be ignored. Further, when caregivers adopted positive coping behaviors that met their needs, it had a positive effect on family need satisfaction [7,8,35]. This suggests that future research should further focus on promoting positive coping behaviors to enhance caregivers’ self-management and adjustment abilities. Among environmental factors, policy support and social attitude were strongly positively correlated with need satisfaction. From the perspective of unequal opportunities [36,37], CWDs and their caregivers are socially vulnerable groups due to certain barriers. Unfriendly social attitudes and inadequate public policy support may further exacerbate unequal opportunities, resulting in the limited utilization of support resources and further restricting the satisfaction of psychological support needs. It can be seen that a favorable social and policy environment is an important distal factor in ensuring that the psychological support needs of caregivers of CWDs are met, and creating a favorable environmental climate demands more effort from society.

### 4.4. The Interaction Effects of Psycho-Behavioral and Social–Environmental Factors

In the field of child care, most existing studies have focused on individual micro-perspectives, while some studies on vulnerable populations have already explored the interaction effects between macro-environmental factors, such as community environment, urban welfare, primary healthcare services, and individual factors. This study examined the interaction from an individual–social interaction perspective, and the results were consistent with the Uses and Gratifications theory [38,39]. Regarding the satisfaction of psychological support needs, there was an interaction effect between caregiver anxiety and social attitude. This may be due to the fact that caregivers in unfriendly social environments are more tolerant and less sensitive to anxiety, making them “tolerate” the effects of anxiety on themselves. Only after improving social attitudes did caregivers start to pay more attention to their own emotions. On the one hand, it is suggested that we need to focus on the social attitudes that caregivers encounter, effectively improve social attitudes, and enhance urban inclusiveness [40] to promote caregivers’ good adaptation and development. On the other hand, we need to focus on caregivers’ negative emotions and strengthen the supply of caregiver support services to provide the material basis for meeting caregivers’ needs.

### 4.5. The Configuration Paths of Psychological Support Needs and Satisfaction

Our findings indicated that various configuration paths were identified as potentially leading to high levels of caregivers’ psychological support needs and low levels of need satisfaction. All paths were classified into three categories, consistent with existing perceptions: the psycho-behavioral-factor-oriented group, the coinfluence-oriented group, and the social–environmental-factor-oriented group. Furthermore, both the SDH model [15] and EST [14] postulate that individuals are influenced by factors at different levels, and in certain cases, these factors may act concurrently to affect individuals. These findings are consistent with the aforementioned theories. In terms of case coverage [41,42], the psycho-behavioral-factor-oriented group was the highest, followed by the coinfluence-oriented group. These two groups were more common in the population. The psycho-behavioral-factor-oriented group was characterized by the presence of either a single emotion or a combination of psychological and behavioral problems. This can be attributed to the prevalence of emotional issues among caregivers. Poor emotional states can give rise to negative behavioral problems [43], and the coexistence of these two factors can lead to unfavorable outcomes. Additionally, although the coverage of the social–environmental-factor-oriented group was low, the factors of policy support and social attitude cannot be ignored. The consistency and coverage of this study were satisfactory. Although the coverage of need satisfaction was relatively low, it was still within the acceptable range. The relatively low coverage might be due to the fact that the cases used for QCA in this study were questionnaire data, and analogous findings have been reported in other questionnaire-based QCA studies [44,45].

### 4.6. Limitations

Our study has several limitations. First, we only considered a few frequently mentioned modifiable factors, which were identified by systematically analyzing the published literature. Consequently, some factors may have been excluded from our research. Second, our study only conducted a simple interaction and configuration analysis of the influencing factors and did not explore the complex influencing mechanisms between the individual and environmental factors and psychological support needs. In addition, considering the lack of mature scales and assessment tools for the measurement of the social environment, this study designed its measurement items based on the literature and the real-life context in China.

## 5. Conclusions

The psychological support need rate among caregivers was high, while the level of need satisfaction was low. There were significant differences in psychological support needs and need satisfaction among different psycho-behavioral and social–environmental characteristics. Regarding need satisfaction, there was an interaction effect between caregiver anxiety and social attitude. Various configuration paths were identified and could be classified into three categories: the psycho-behavioral-factor-oriented group, the coinfluence-oriented group, and the socio–environmental-factor-oriented group. Therefore, in the future, it is necessary to provide appropriate demand-driven psychological support services. At the same time, it is recommended to further focus on caregiver anxiety, integrate resources from various stakeholders, improve social attitudes, promote policy implementation, and establish feasible and effective intervention programs to promote the well-being and development of CWDs and their caregivers.

## Figures and Tables

**Table 1:** Characteristics of participants.

Dimension	Variable	Variable Category	N	%
Children individual characteristics (control variables)	Gender	male	230	63.36
		female	133	36.64
	Age group (years)	[0, 6)	202	55.65
		[6, 12)	122	33.61
		[12, 18)	39	10.74
	Disability type	developmental delay	39	10.74
		visual disability	24	6.61
		hearing disability	57	15.70
		speech disability	25	6.89
		physical disability	83	22.87
		intellectual disability	36	9.92
		mental disability	53	14.60
		multiple disabilities	46	12.67
	Cause of disability	congenital	155	42.70
		acquired	208	57.30
	Other diseases	yes	62	17.08
		no	301	82.92
	Emotional stability	very unstable	7	1.90
		relatively unstable	48	13.20
		general	86	23.70
		relatively stable	162	44.60
		very stable	60	16.50
Caregiver individual characteristics (control variables)	Caregiver Role	father	98	27.00
		mother	265	73.00
	Age group (years) *	≤30	38	10.53
		≤40	221	61.22
		>40	102	28.25
	Education	below bachelor degree	166	45.70
		bachelor degree and above	197	54.30
	Employment status	no job	117	32.23
		part-time	44	12.12
		full-time	202	55.65
	Caregiving time (years) †	≤5	214	59.94
		5–10	95	26.61
		≥10	48	13.45
Family characteristics (control variables)	Core Family population (person)	≤3	133	36.64
		4–5	180	49.59
		6	50	13.77
	Household income monthly (CNY) ‡	<5000	136	37.78
		5000–9999	102	28.33
		≥10,000	122	33.89
	Household expenditure monthly (CNY) §	≤10,000	163	46.70
		10,001–20,000	128	36.68
		>20,000	58	16.62
Psycho-behavioral factors	Anxiety	anxiety symptoms	130	35.81
		no symptoms of anxiety	233	64.19
	Mental	poor mental health	236	65.01
		good mental health	127	34.99
	Coping behavior †	negative behavior	111	31.01
		positive behavior	247	68.99
Social–environmental factors	Policy support *	dissatisfaction	92	25.48
		fair	183	50.69
		satisfaction	86	23.82
	Social attitude *	unfriendliness	124	34.35
		fair	142	39.34
		friendliness	95	26.32

* 2 dates missing; † 5 dates missing; ‡ 3 dates missing; § 14 dates missing.

**Table 2:** Univariate analysis of influencing factors.

Dimension	Factor	Rate		Satisfaction	
		Statistic	*p*	Statistic	*p*
Individual and family characteristics					
Children	Gender	0.054	0.817	1.428	0.232
	Age	3.346	0.188	0.781	0.677
	Disability type	9.596	0.213	26.418	<0.001 **
	Cause of disability	0.581	0.446	4.245	0.039 *
	Other diseases	2.226	0.136	1.793	0.181
	Emotional stability	25.825	<0.001 **	7.546	0.110
Caregiver	Caregiver Role	1.884	0.170	5.475	0.019 *
	Age	5.416	0.067	0.868	0.648
	Education	1.380	0.240	0.551	0.458
	Employment status	2.288	0.319	3.594	0.166
	Caregiving time	0.691	0.708	0.881	0.644
Family	Core family population	0.760	0.684	2.917	0.233
	Household income monthly	0.685	0.710	4.374	0.112
	Household expenditure monthly	1.475	0.478	4.172	0.124
Modifiable psycho-behavioral and social–environmental factors					
Psycho-behavioral factors	Anxiety	16.620	<0.001 **	18.252	<0.001 **
	Mental health	7.063	0.008 *	−5.389	0.020 *
	Coping behavior	0.125	0.900	−2.332	0.020 *
Social–environmental factors	Policy support	7.895	0.019 *	32.566	<0.001 **
	Social attitude	0.684	0.710	40.497	<0.001 **

* *p* < 0.05; ** *p* < 0.01.

**Table 3:** Multivariable logistic regression model for psychological support needs.

Dimension	Factor	Model 1					Model 2					Model 3				
		*p*	β	OR	95%CI		*p*	β	OR	95%CI		*p*	β	OR	95%CI	
Individual and family characteristics	Emotional stability	0.019 *	−0.398	0.671	0.481	0.938	0.024 *	−0.390	0.677	0.483	0.949	0.028 *	−0.381	0.683	0.486	0.960
Psycho-behavioral factors	Anxiety	0.002 **	0.169	1.185	1.062	1.321	0.003 **	0.167	1.182	1.059	1.319	0.002 **	0.178	1.194	1.065	1.340
	Mental health	0.200	−0.015	0.985	0.962	1.008	0.204	−0.015	0.985	0.962	1.008	0.225	−0.015	0.985	0.962	1.009
Social–environmental factors	Policy support						0.760	−0.064	0.938	0.622	1.415	0.725	0.092	1.096	0.656	1.832
Interaction items	Anxiety × policy support											0.334	0.075	1.077	0.926	1.254
	Mental health × policy support											0.861	−0.003	0.997	0.965	1.031
Cox and Snell R^2^		0.080					0.080					0.083				
Nagelkerke R^2^		0.132					0.133					0.137				

* *p* < 0.05; ** *p* < 0.01.

**Table 4:** Multivariable logistic regression model for the satisfaction of psychological support needs.

Dimension	Factor		Model 1					Model 2					Model 3				
			*p*	β	OR	95%CI		*p*	β	OR	95%CI		*p*	β	OR	95%CI	
Individual and family characteristics	Disability type	developmental delay	0.032 *					0.165					0.111				
		visual disability	0.014 *	1.595	4.929	1.387	17.518	0.058	1.295	3.649	0.958	13.896	0.052	1.364	3.910	0.991	15.424
		hearing disability	0.052	0.935	2.548	0.991	6.550	0.432	0.404	1.498	0.546	4.108	0.434	0.415	1.514	0.536	4.275
		speech disability	0.098	0.972	2.643	0.837	8.349	0.155	0.876	2.402	0.719	8.026	0.158	0.888	2.430	0.708	8.345
		physical disability	0.109	0.761	2.141	0.845	5.425	0.299	0.511	1.667	0.636	4.370	0.274	0.553	1.739	0.646	4.683
		intellectual disability	0.720	−0.219	0.803	0.243	2.655	0.778	−0.179	0.836	0.241	2.900	0.638	−0.307	0.736	0.205	2.644
		mental disability	0.552	0.298	1.347	0.505	3.592	0.214	0.651	1.917	0.688	5.345	0.177	0.714	2.041	0.724	5.754
		multiple disabilities	0.873	−0.087	0.917	0.314	2.677	0.497	−0.396	0.673	0.215	2.109	0.429	−0.472	0.623	0.193	2.013
	Cause of disability	acquired	0.267	0.287	1.333	0.802	2.213	0.320	0.273	1.314	0.767	2.251	0.345	0.266	1.304	0.751	2.263
	Caregiver role	mother	0.196	0.361	1.435	0.830	2.479	0.173	0.407	1.502	0.837	2.698	0.210	0.387	1.472	0.804	2.698
Psycho-behavioral factors	Anxiety		0.041 *	−0.072	0.931	0.869	0.997	0.078	−0.064	0.938	0.874	1.007	0.146	−0.052	0.950	0.886	1.018
	Mental health		0.179	0.016	1.016	0.993	1.040	0.192	0.016	1.017	0.992	1.042	0.111	0.021	1.021	0.995	1.048
	Coping behavior		0.413	0.012	1.012	0.983	1.043	0.527	0.010	1.010	0.979	1.041	0.455	0.012	1.012	0.981	1.043
Social–environmental factors	Policy support							0.010 *	0.568	1.764	1.148	2.711	0.009 **	0.601	1.823	1.164	2.855
	Social attitude							<0.001 **	0.684	1.982	1.362	2.886	0.002 **	0.635	1.887	1.264	2.816
Interaction items	Anxiety × policy support												0.782	0.016	1.016	0.909	1.135
	Anxiety × social attitude												0.018 *	−0.134	0.874	0.783	0.977
	Mental health × policy support												0.374	0.017	1.017	0.980	1.056
	Mental health × social attitude												0.155	−0.025	0.975	0.942	1.009
	Coping behavior × policy support												0.258	0.026	1.026	0.981	1.072
	Coping behavior × social attitude												0.216	−0.025	0.975	0.936	1.015
Cox and Snell R^2^			0.118					0.200					0.222				
Nagelkerke R^2^			0.162					0.276					0.305				

* *p* < 0.05; ** *p* < 0.01.

**Table 5:** Analysis of necessary conditions.

Condition Variables	Psychological Support Need		Satisfaction of Psychological Support Need	
	Consistency	Coverage	Consistency	Coverage
MH	0.679	0.863	0.688	0.695
~MH	0.321	0.760	0.312	0.563
AS	0.402	0.937	0.447	0.800
~AS	0.598	0.766	0.553	0.561
CB	0.311	0.829	0.363	0.709
~CB	0.689	0.826	0.637	0.617
PS	0.280	0.912	0.330	0.798
~PS	0.720	0.798	0.670	0.593
SA	0.345	0.829	0.433	0.782
~SA	0.655	0.826	0.567	0.573

Note: “~” denotes the absence of a condition. For instance, MH represents “poor mental health”, and ~MH represents “good mental health”.

**Table 6:** Configuration path of psychological support needs and need satisfaction.

Conditional Combination	Psychological Support Need						Satisfaction of Psychological Support Need				
	Psycho-Behavioral-Factor-Oriented Group			Coinfluence-Oriented Group		Social–Environmental-Factor-Oriented Group	Psycho-Behavioral-Factor-Oriented Group		Coinfluence-Oriented Group		Social–Environmental-Factor-oriented Group
	Path 1	Path 2	Path 3	Path 4	Path 5	Path 6	Path 1	Path 2	Path 3	Path 4	Path 5
Poor mental health (MH)	●		▲	▲	▲	○		▲	▲		○
Anxiety symptoms (AS)		●	●	●		△	●	▲	●	○	△
Negative coping behavior (CB)	○		●		●		●	▲	○	●	△
Dissatisfaction with policy support (PS)		△		●	●	●	○	△	●	●	●
Unfriendly social attitudes (SA)	○	○				●	△			●	○
Consistency	0.871	0.939	0.949	0.973	0.960	0.875	0.905	0.957	0.905	1.000	1.000
Raw coverage	0.365	0.209	0.125	0.122	0.081	0.047	0.088	0.102	0.088	0.051	0.019
Unique coverage	0.209	0.037	0.027	0.041	0.030	0.047	0.023	0.037	0.088	0.051	0.019
Overall coverage	0.645						0.284				
Overall consistency	0.901						0.938				

Note: “●” denotes the presence of a core condition; “○” denotes the absence of a core condition; “▲” denotes the presence of a peripheral condition; “△” denotes the absence of a peripheral condition; a space means that the factor has no effect on the outcome.

## Data Availability

Data cannot be shared publicly because of the privacy implications but are available upon reasonable request. Data requests may be sent to the corresponding authors.
